# Transforming Medical Paradigms: A Cutting-Edge Review of Genomic and Robotic Medical and Surgical Approaches in the Battle Against Diabetes, Hypertension, and Cardiovascular Issues

**DOI:** 10.7759/cureus.46998

**Published:** 2023-10-13

**Authors:** Yousaf Tanveer, Sailakshmn Sanipini, Rafeef Khleif, Tamar Tsenteradze, Abubakar Gapizov, Han Grezenko, Maryam Affaf, Ali M Abdelaziz, Abdur Rehman, Umar Zia, Huda Jama, Abdullah Shehryar, Syed Naveed Mohsin, Chukwuyem Ekhator, Rehman Khan

**Affiliations:** 1 Urology, Craigavon Area Hospital, Craigavon, GBR; 2 Medical School, Xavier University School of Medicine, Oranjestad, ABW; 3 General Surgery, Tbilisi State Medical Univerity, Tbilisi, GEO; 4 Cardiology, Tbilisi State Medical Univerity, Tbilisi, GEO; 5 Internal Medicine, Tbilisi State Medical Univerity, Tbilisi, GEO; 6 General Surgery, American University of Antigua, Saint George, ATG; 7 Translational Neuroscience, Barrow Neurological Institute, Phoenix, USA; 8 Internal Medicine, Women’s Medical and Dental College, Abbotabad, PAK; 9 Internal Medicine, Alexandria University Faculty of Medicine, Alexandria, EGY; 10 Surgery, Mayo Hospital, Lahore, PAK; 11 Internal Medicine, Khyber Medical University, Peshawar, PAK; 12 Internal Medicine, Nishtar Medical University, Multan, PAK; 13 Internal Medicine, Allama Iqbal Medical College, Lahore, PAK; 14 General Surgery, Cavan General Hospital, Cavan, IRL; 15 Neuro-Oncology, New York Institute of Technology, College of Osteopathic Medicine, Old Westbury, USA; 16 Internal Medicine, Mayo Hospital, Lahore, PAK

**Keywords:** diabetes mellitus, future management, clinical genomics, cva, hypertension

## Abstract

This article provides an in-depth review of the current state of management for diabetes, hypertension, and cardiovascular disease, focusing on advancements from genomics to robotics. It explores the role of genomic markers in personalized medicine, offering tailored treatment options for these chronic conditions. The article also examines the efficacy of various pharmacological and surgical interventions, including bariatric surgery for diabetes and device-based treatments for hypertension. A comparative analysis is presented to evaluate the cost-effectiveness and patient outcomes between medical and surgical approaches. The review concludes that while personalized medicine and minimally invasive surgical techniques show promise, more high-quality comparative research is needed. The ultimate goal is to integrate these emerging technologies within a framework of evidence-based medicine to improve patient outcomes and health equity.

## Introduction and background

Interconnected medical conditions, such as diabetes, hypertension, and cardiovascular disease (CVD), have a profound impact on global health. Type 2 diabetes, in particular, is characterized by chronic hyperglycemia and insulin resistance, which lead to vascular complications. Several underlying mechanisms contribute to these issues, including the formation of advanced glycation end products (AGEs), oxidative stress, and the activation of the AGE-receptor for AGEs (RAGE) axis [1].

CVD remains the leading cause of morbidity and mortality among individuals with diabetes. While rigorous glycemic control has its benefits, it is not without drawbacks [2]. Hypertension and dyslipidemia are often comorbid with type 2 diabetes and serve as significant risk factors for atherosclerotic cardiovascular disease (ASCVD). Effective management of these risk factors can halt or slow the progression of ASCVD [3].

The relationship between diabetes and hypertension is further complicated by shared risk factors such as endothelial dysfunction, vascular inflammation, atherosclerosis, and obesity. These factors exacerbate the cardiovascular complications associated with diabetes. Notably, the prevalence of hypertension is higher among individuals with diabetes compared to those without the condition [4].

This narrative review aims to offer a comprehensive overview of the latest medical and surgical approaches for treating diabetes, hypertension, and cardiovascular events. This encompasses everything from genomic discoveries to advancements in robotic technologies. The objectives are multi-faceted: to review recent genomic studies on the genetic etiology and biomarkers of cardiovascular diseases, hypertension, and diabetes; to summarize current pharmaceutical treatments for glycemic, blood pressure, and cardiovascular event control; to describe significant advancements in surgical methods; to consider the growing roles of robotic surgery, artificial intelligence, and digital health; to identify areas for further research and innovation; and to offer recommendations for personalized treatment strategies aimed at improving patient outcomes.

## Review

Genomics and personalized medicine

Genomic Markers in Diabetes

TCF7L2: Variants in the Transcription Factor 7-Like 2 (TCF7L2) gene represent the most significant genetic risk factor for type 2 diabetes, as evidenced by a meta-analysis involving over 30,000 individuals [5]. These variants are linked to impaired insulin secretion, suggesting that patients carrying this genetic marker may benefit from early initiation of insulin therapy.

PPARG: Polymorphisms in the Peroxisome Proliferator-Activated Receptor Gamma (PPARG) gene, a nuclear receptor crucial for glucose and lipid metabolism, have a notable impact on insulin sensitivity. Specifically, the P12A variant offers protective effects against the progression of type 2 diabetes [6]. Therefore, medications like thiazolidinediones, which target PPARG, may be particularly effective for patients with this variant.

KCNJ11: Variants in the Potassium Inwardly Rectifying Channel, Subfamily J, Member 11 (KCNJ11) gene, which encodes a pancreatic beta-cell potassium channel, influence insulin secretion [7]. Patients with these variants tend to respond more favorably to sulfonylurea drugs that target the encoded KATP channel, guiding the selection of these therapeutic agents.

TCF2: Polymorphisms in the Transcription Factor 2 (TCF2) gene, which regulates proinsulin processing, elevate the risk of type 2 diabetes by impairing insulin secretion [8]. For patients with TCF2 abnormalities, enhancing endogenous insulin secretion through both pharmaceutical and lifestyle interventions appears to be the optimal management strategy.

CDKAL1: Variants in the CDK5 Regulatory Subunit Associated Protein 1-Like 1 (CDKAL1) gene, involved in cell cycle regulation, compromise beta-cell function and increase the risk of type 2 diabetes [9]. Patients with these variants may benefit more from therapies aimed at improving beta-cell function and mass, such as GLP-1 agonists.

IGF2BP2: A specific variant in the Insulin-Like Growth Factor 2 mRNA Binding Protein 2 (IGF2BP2) gene is linked to both insulin resistance and impaired insulin secretion [10]. Patients with this variant tend to have a poor response to metformin, suggesting the need for alternative first-line medications.

Genomic Markers in Hypertension

ADD1: Variants in the Adducin 1 (ADD1) gene, which plays a role in renal sodium reabsorption, have been linked to an elevated risk of hypertension [11]. Studies have shown that sodium restriction can significantly lower blood pressure in individuals carrying these ADD1 variants.

AGT: Polymorphisms in the Angiotensinogen (AGT) gene, responsible for encoding a precursor of the vasoconstrictor angiotensin II, are associated with a predisposition to hypertension [12]. Patients with these AGT variants may particularly benefit from angiotensin receptor blockers like losartan, which directly counteract the effects of elevated angiotensin II levels.

CYP11B2: Variants in the Cytochrome P450 Family 11 Subfamily B Member 2 (CYP11B2) gene, which encodes aldosterone synthase, play a role in blood pressure regulation [13]. Mineralocorticoid receptor antagonists, such as spironolactone, may be especially effective in treating hypertension in patients with CYP11B2 variants that affect aldosterone synthesis.

WNK1: Abnormalities in the expression of WNK Lysine Deficient Protein Kinase 1 (WNK1) can lead to monogenic hypertension through their effects on renal electrolyte transport [14]. Hypertension linked to WNK1 appears to be uniquely responsive to thiazide diuretics due to the associated electrolyte transport abnormalities.

NPPA: The rs5068 variant in the Natriuretic Peptide A (NPPA) gene, which encodes atrial natriuretic peptide, offers protective effects against hypertension [15]. This NPPA rs5068 variant is associated with a reduced need for antihypertensive medications, guiding a more conservative approach to prescribing.

NEDD4L: Variants in the Neural Precursor Cell Expressed, Developmentally Down-Regulated 4-Like (NEDD4L) gene influence blood pressure by altering sodium reabsorption [16]. Sodium-wasting diuretics like amiloride may be effective in normalizing sodium reabsorption in patients with these NEDD4L variants.

Genomic Markers in Cardiovascular Events

9p21: Variants in the 9p21 genomic region are associated with an increased risk for conditions such as myocardial infarction, coronary artery disease, and aneurysms, primarily through their effects on vascular cell proliferation [17]. Patients carrying these 9p21 variants may benefit more from aggressive management of modifiable risk factors like LDL cholesterol and blood pressure.

KIF6: The KIF6 719Arg variant in the Kinesin Family Member 6 gene is linked to an elevated risk of coronary heart disease [18]. This particular KIF6 variant has been shown to respond more effectively to statin therapy for the prevention of coronary heart disease.

LPA: Variants in the Lipoprotein(a) gene (LPA) influence Lp(a) levels and the risk of atherosclerotic cardiovascular disease [19]. For individuals with these LPA variants, Lipoprotein apheresis may be a more effective treatment for lowering Lp(a) levels and cardiovascular risk when conventional drug therapies are ineffective.

PCSK9: Loss-of-function mutations in the Proprotein Convertase Subtilisin/Kexin Type 9 (PCSK9) gene result in lower LDL cholesterol levels and offer protection against cardiovascular events [20]. These PCSK9 loss-of-function mutations indicate a better response to PCSK9 inhibitor drugs like alirocumab and evolocumab for lowering LDL cholesterol.

APOA5: Rare variants in the Apolipoprotein A5 (APOA5) gene disrupt triglyceride metabolism and increase susceptibility to ischemic heart disease [21]. These APOA5 variants are associated with a poorer response to standard triglyceride-lowering therapies, suggesting the need for newer agents like icosapent or pemafibrate.

MTHFR: The MTHFR 677C>T variant impacts folate metabolism and increases susceptibility to vascular diseases [22]. Patients with the MTHFR 677TT variant may benefit from increased folate intake to lower homocysteine levels and reduce vascular risk.

Medical management of diabetes mellitus

Pharmacological Treatment

Metformin: Metformin stands as the preferred initial oral medication for managing type 2 diabetes, owing to its proven efficacy, safety, and cost-effectiveness [23].

Sulfonylureas: Second-line agents like glipizide and glimepiride fall under the category of sulfonylureas, which function by stimulating insulin secretion and are commonly used in the treatment of type 2 diabetes.

Thiazolidinediones: Drugs such as pioglitazone and rosiglitazone belong to the thiazolidinedione class and work by improving insulin sensitivity. They may also contribute to the durability of glycemic control.

GLP-1 receptor agonists: Injectable GLP-1 analogs, including liraglutide and semaglutide, serve to lower glucose levels and promote weight loss, offering a dual benefit.

SGLT2 inhibitors: SGLT2 inhibitors, such as empagliflozin and canagliflozin, function by reducing glucose reabsorption in the kidneys, thereby aiding in glycemic control.

Insulin therapy: The administration of exogenous insulin is indispensable for the management of type 1 diabetes and often becomes necessary in the later stages of type 2 diabetes.

Lifestyle Modification

Physical activity: It is recommended that individuals engage in at least 150 minutes of moderate-intensity aerobic activity per week, supplemented by resistance training two to three days per week. This regimen aims to enhance glycemic control, cardiovascular health, weight management, and overall well-being [24].

Weight management: A modest weight loss of 5-10%, achieved through caloric restriction and increased physical activity, can yield significant improvements in hyperglycemia and other cardiovascular risk factors, particularly in overweight or obese patients.

Diet: Medical nutrition therapy is advised for optimizing glycemic control, lipid profiles, and blood pressure. This involves limiting the intake of refined carbohydrates and added sugars while emphasizing high-fiber foods, lean proteins, and healthy fats.

Smoking cessation: All patients who smoke should be strongly encouraged to quit, given the elevated cardiovascular risks associated with both smoking and diabetes.

Stress management: The adoption of healthy coping strategies for stress, such as relaxation techniques, can positively impact diabetes management.

Medical management of hypertension

Pharmacological Treatment

Thiazide diuretics: Thiazide diuretics, such as chlorthalidone and hydrochlorothiazide, are often the preferred initial agents for most patients. Their preference is based on their proven efficacy, cost-effectiveness, and favorable outcomes data [25].

ACE inhibitors: Angiotensin-converting enzyme (ACE) inhibitors, including lisinopril and enalapril, serve as effective first-line options. They are particularly beneficial for patients with diabetes or heart failure.

Angiotensin receptor blockers: Angiotensin receptor blockers (ARBs) like losartan and valsartan offer benefits similar to ACE inhibitors but generally come with fewer side effects.

Calcium channel blockers: Non-dihydropyridine calcium channel blockers (CCBs), such as diltiazem and verapamil, are useful as add-on medications. They are especially beneficial for older patients who may require additional blood pressure control.

Beta-blockers: Beta-blockers, like metoprolol, are effective in reducing blood pressure but generally have more limited effects on long-term outcomes compared to other classes of antihypertensive medications.

Lifestyle Modification

Weight loss: Reducing body weight has been shown to lower blood pressure in overweight and obese patients diagnosed with hypertension.

Dietary sodium reduction: Limiting daily sodium intake to no more than 2400 mg is recommended for achieving optimal blood pressure reduction.

DASH diet: The Dietary Approaches to Stop Hypertension (DASH) diet, which is rich in fruits, vegetables, nuts, and low-fat dairy while being low in fats and sweets, has demonstrated antihypertensive effects [26].

Physical activity: Engaging in aerobic exercise for a minimum of 90-150 minutes per week has been shown to lower blood pressure and enhance the effectiveness of pharmacological treatments.

Moderation of alcohol: For those who consume alcohol, limiting intake to a maximum of two drinks daily for men and one drink for women can contribute to blood pressure reduction.

Smoking cessation: Quitting smoking not only lowers blood pressure but also eliminates the additional cardiovascular risks associated with smoking.

Stress management: Employing stress management techniques, such as meditation, may assist in controlling blood pressure levels.

Medical management of cardiovascular events

Pharmacological Treatment

Antiplatelet agents: Antiplatelet medications, such as aspirin and P2Y12 inhibitors (e.g., clopidogrel, prasugrel), play a crucial role in preventing recurrent ischemic events in patients with atherosclerotic disease [27].

Beta-blockers: Beta-blockers serve as a standard therapy for treating heart failure and reducing mortality following a myocardial infarction.

ACE inhibitors/ARBs: Angiotensin-converting enzyme (ACE) inhibitors like enalapril and angiotensin receptor blockers (ARBs) like losartan are frontline therapies for managing heart failure and post-myocardial infarction care.

Statins: High-intensity statin therapy, including drugs like atorvastatin and rosuvastatin, effectively lowers LDL cholesterol levels and reduces the risk of recurrent cardiovascular events.

Anticoagulants: Anticoagulant medications are indispensable for managing thrombotic events such as deep vein thrombosis (DVT), pulmonary embolism (PE), and atrial fibrillation-related strokes.

Nitrates: Nitrates like isosorbide mononitrate alleviate angina symptoms in patients with coronary artery disease through vasodilation.

Calcium channel blockers: Non-dihydropyridine calcium channel blockers (CCBs), such as diltiazem, are effective in managing heart rate in atrial fibrillation and relieving angina symptoms.

Lifestyle Modification

Smoking cessation: Quitting smoking is crucial for lowering the risk of recurrent cardiovascular events and mortality, especially in patients with a history of cardiovascular disease (CVD).

Weight management: A gradual weight loss of 5-10% in overweight or obese patients can significantly reduce cardiovascular risk factors such as blood pressure, lipid levels, and glucose levels.

Dietary pattern: Adopting a diet rich in fruits, vegetables, whole grains, lean proteins, nuts, legumes, and healthy oils - akin to the Mediterranean diet - offers protective benefits against CVD.

Sodium reduction: Limiting daily sodium intake to between 1500 and 2000 mg can effectively lower blood pressure and subsequently reduce the risk of CVD.

Physical activity: Engaging in 150-300 minutes of moderate exercise per week, such as brisk walking, contributes to weight loss, glucose control, blood pressure reduction, and overall cardiovascular health.

Stress management: Mind-body practices like meditation, yoga, tai chi, or breathing exercises may have beneficial effects on cardiovascular health.

Surgical interventions for diabetes mellitus

Bariatric Surgery

Bariatric surgery (e.g., Roux-en-Y gastric bypass, sleeve gastrectomy, biliopancreatic diversion) is a notable solution for obesity-related type 2 diabetes [28]. Controlled trials prove it surpasses medical therapy by achieving better glycemic control, remission, weight loss, and cardiovascular benefits. Yet, challenges encompass perioperative risks, nutritional deficiencies, and lifestyle changes, with declining remission rates over time. Financial and accessibility barriers limit wider use. While effective for suitable candidates, multidisciplinary care is essential due to individual complications and needs [29]. More research on long-term outcomes is needed.

Pancreatic Transplant

Pancreatic transplantation restores insulin production in type 1 diabetes through the whole pancreas or islet cell transplant [30]. Success achieves insulin independence and improves glycemic control, reducing complications and enhancing life quality. Challenges include immunosuppression, surgical risks, organ scarcity, and graft failure risks. Limited eligibility exists, despite effectiveness in restoring euglycemia without insulin. Risks, rejection, and limitations restrict wider use, though organ advancements may broaden feasibility for more patients [31].

Surgical interventions for hypertension

Renal Denervation

Renal denervation lowers blood pressure by ablating renal sympathetic nerves in hypertension patients. Trials reveal blood pressure reduction, aiding resistant hypertension cases and even reducing medication needs. Yet, effectiveness varies across trials, some showing no significant benefit. The procedure remains investigational with unclear long-term effects and safety concerns like renal artery stenosis [32]. Renal denervation holds promise for hypertension management, but more data on patient selection, techniques, and long-term outcomes are needed to define its role definitively.

Carotid Baroreceptor Stimulation

Carotid baroreceptor stimulation uses an implantable device to activate the carotid baroreflex, reducing blood pressure by inhibiting vasoconstriction and sympathetic activity. Studies show sustained blood pressure reduction and safety, potentially lowering medication needs. Surgical risks exist, and not all patients respond. Long-term durability and impact on cardiovascular outcomes remain uncertain while costs are high. Despite the promise of treating resistant hypertension, clinical trials are necessary to define its role [33].

Surgical interventions for cardiovascular events

Coronary Artery Bypass Grafting (CABG)

Coronary artery bypass grafting (CABG) surgically bypasses arterial blockages, restoring blood flow to the heart. CABG reduces mortality and heart attack risk, especially in complex cases, offering more lasting revascularization than PCI. Yet, risks include bleeding, arrhythmias, infection, and graft issues, with prolonged recovery. Higher costs than PCI exist. CABG suits complex cases where PCI isn't viable, backed by strong evidence for event reduction [34]. Surgery's invasiveness must balance individual risk factors carefully.

Heart Transplant

Heart transplantation replaces a failing heart with a healthy donor heart, offering gold-standard treatment for end-stage heart failure. It enhances symptoms, exercise capacity, and life quality in select refractory patients. Challenges encompass organ scarcity, surgical risks, high immunosuppression costs, and potential rejection or graft vasculopathy [35]. Rigorous candidate screening is crucial. Heart transplantation remains lifesaving for severe heart failure, yet availability limits its use. Better donor allocation and graft failure management could broaden feasibility.

Robotics and emerging technologies

Robotic Surgery in Diabetes

Robotic systems like da Vinci enable minimally invasive bariatric and metabolic procedures for diabetes linked to obesity. Compared to open surgery, they bring benefits such as less blood loss, shorter hospital stays, and similar outcomes in weight loss and diabetes remission. Yet, drawbacks exist: high costs, limited availability, and longer operation times. Specialized training is needed due to a lack of tactile feedback. Risks involve complications like bleeding, infection, leaks, and potential device-related issues. Despite challenges, robotic bariatric surgery holds potential for diabetes management, but high costs hinder wider use. Research is vital to establish safety and long-term efficacy [36].

Robotic Surgery in Hypertension

Robotic platforms are used for minimally invasive hypertension treatments like renal denervation and baroreceptor activation. They offer the benefits of smaller incisions, quicker recovery, and improved visualization [37]. Challenges include costs, limited availability, learning curve, and unproven efficacy. General risks like bleeding and infection apply, alongside device malfunction and stimulation issues. Despite hurdles, robotic systems show potential for precise, minimally invasive hypertension procedures, though utility remains uncertain due to costs and expertise demands.

Robotic Surgery in Cardiovascular Events

Robotic platforms enhance cardiovascular procedures like CABG and mitral valve repair, offering benefits such as minimal invasiveness, reduced pain, and quicker recovery [38]. Challenges include learning curve, costs, and limited data on long-term effectiveness. Similarities exist in clinical outcomes like mortality and stroke risk, while technical issues like anastomoses difficulties and device malfunction persist. Though enabling minimally invasive complexity, adoption is hindered by costs, lack of superiority proof, and expertise demands.

Prospects in the field of robotic surgeries for diabetes, hypertension, and cardiovascular diseases

Advancements in robotic surgery enhance diabetes and cardiovascular treatment. Robotics improve bariatric surgery, aiding diabetes remission. Innovations in catheter navigation and renal systems for hypertension promise safer, fully robotic procedures. Complex cardiovascular surgeries will benefit from enhanced robotic platforms and training [39]. The future holds minimally invasive metabolic and cardiovascular treatments, though comparative data against traditional methods is crucial for acceptance and implementation.

In the realm of treating diabetes, hypertension, and cardiovascular events, a comparative analysis between medical and surgical approaches offers valuable insights into cost-effectiveness and patient outcomes. For diabetes, although bariatric surgery entails higher upfront costs compared to medications, it proves to be more cost-effective in the long run. This surgical approach achieves superior glycemic control and higher rates of diabetes remission [40]. On the other hand, hypertension is generally better managed through medications, which serve as the first-line treatment. These medications are more cost-effective than emerging surgical techniques like renal denervation, which currently lack conclusive data to support their clinical benefits [41]. When it comes to cardiovascular events, revascularization procedures like coronary artery bypass grafting (CABG) may be costly initially but yield improved outcomes compared to medical therapy alone, especially for high-risk patients with multi-vessel disease. In summary, diabetes currently appears to be the most amenable to surgical intervention while hypertension is better managed medically. For cardiovascular events, the optimal treatment strategy is guided by the individual patient's clinical context. Further research is needed to provide more comprehensive comparative data.

## Conclusions

This review provides a comprehensive overview of the current advancements in the management of diabetes, hypertension, and cardiovascular disease, ranging from genomics to robotics. Personalized medicine, guided by genomic profiling, is increasingly influencing treatment choices, especially in diabetes and cardiovascular disease. However, for genomics to fully realize its potential, more research is needed to identify additional variants and establish evidence-based guidelines. Bariatric surgery has shown promise in treating diabetes while pharmacological approaches remain the cornerstone for hypertension management. Device-based interventions and surgical revascularization offer new avenues but require further validation through randomized trials.

The overarching theme is a shift toward more integrative, individualized, and minimally invasive treatments, supported by innovations in molecular genetics and robotics. However, the integration of these emerging tools must be rooted in evidence-based medicine, patient-centered care, and equitable access. As technology continues to advance, high-quality comparative research is essential for translating these innovations into meaningful improvements in patient outcomes and health equity. This is particularly crucial given the increasing global burden of chronic diseases like diabetes, hypertension, and cardiovascular disease.

## References

[1] Petrie JR, Guzik TJ, Touyz RM (2018). Diabetes, hypertension, and cardiovascular disease: clinical insights and vascular mechanisms. Can J Cardiol.

[2] Schmidt AM (2019). Diabetes and cardiovascular disease. Emerging therapeutic approaches. Arterioscler Thromb Vasc Biol.

[3] (2020). 10. Cardiovascular disease and risk management: standards of medical care in diabetes—2020. Diabetes Care.

[4] Unger T, Borghi C, Charchar F (2020). 2020 International Society of Hypertension global hypertension practice guidelines. Hypertension.

[5] Grant SF, Thorleifsson G, Reynisdottir I (2006). Variant of transcription factor 7-like 2 (TCF7L2) gene confers risk of type 2 diabetes. Nat Genet.

[6] Altshuler D, Hirschhorn JN, Klannemark M (2000). The common PPARgamma Pro12Ala polymorphism is associated with decreased risk of type 2 diabetes. Nat Genet.

[7] Nielsen EM, Hansen L, Carstensen B (2003). The E23K variant of Kir6.2 associates with impaired post-OGTT serum insulin response and increased risk of type 2 diabetes. Diabetes.

[8] Lyssenko V, Lupi R, Marchetti P (2007). Mechanisms by which common variants in the TCF7L2 gene increase risk of type 2 diabetes. J Clin Invest.

[9] Steinthorsdottir V, Thorleifsson G, Reynisdottir I (2007). A variant in CDKAL1 influences insulin response and risk of type 2 diabetes. Nat Genet.

[10] Cauchi S, Meyre D, Dina C (2006). Transcription factor TCF7L2 genetic study in the French population: expression in human beta-cells and adipose tissue and strong association with type 2 diabetes. Diabetes.

[11] Manunta P, Lavery G, Lanzani C (2008). Physiological interaction between alpha-adducin and WNK1-NEDD4L pathways on sodium-related blood pressure regulation. Hypertension.

[12] Jeunemaitre X (2008). Genetics of the human renin angiotensin system. J Mol Med (Berl).

[13] Lim PO, Macdonald TM, Holloway C (2002). Variation at the aldosterone synthase (CYP11B2) locus contributes to hypertension in subjects with a raised aldosterone-to-renin ratio. J Clin Endocrinol Metab.

[14] Newhouse SJ, Wallace C, Dobson R (2005). Haplotypes of the WNK1 gene associate with blood pressure variation in a severely hypertensive population from the British Genetics of Hypertension study. Hum Mol Genet.

[15] Newton-Cheh C, Larson MG, Vasan RS (2009). Association of common variants in NPPA and NPPB with circulating natriuretic peptides and blood pressure. Nat Genet.

[16] Ji W, Foo JN, O'Roak BJ (2008). Rare independent mutations in renal salt handling genes contribute to blood pressure variation. Nat Genet.

[17] McPherson R, Tybjaerg-Hansen A (2016). Genetics of coronary artery disease. Circ Res.

[18] Shiffman D, O'Meara ES, Bare LA (2008). Association of gene variants with incident myocardial infarction in the Cardiovascular Health Study. Arterioscler Thromb Vasc Biol.

[19] Clarke R, Peden JF, Hopewell JC (2009). Genetic variants associated with Lp(a) lipoprotein level and coronary disease. N Engl J Med.

[20] Cohen JC, Boerwinkle E, Mosley TH Jr, Hobbs HH (2006). Sequence variations in PCSK9, low LDL, and protection against coronary heart disease. N Engl J Med.

[21] Zhou J, Xu L, Huang RS (2013). Apolipoprotein A5 gene variants and the risk of coronary heart disease: a case‑control study and meta‑analysis. Mol Med Rep.

[22] Kluijtmans LA, Whitehead AS (2001). Methylenetetrahydrofolate reductase genotypes and predisposition to atherothrombotic disease; evidence that all three MTHFR C677T genotypes confer different levels of risk. Eur Heart J.

[23] American Diabetes Association Professional Practice Committee (2022). 9. Pharmacologic approaches to glycemic treatment: standards of medical care in diabetes-2022. Diabetes Care.

[24] American Diabetes Association Professional Practice Committee (2022). 5. Facilitating behavior change and well-being to improve health outcomes: standards of medical care in diabetes-2022. Diabetes Care.

[25] Whelton PK, Carey RM, Aronow WS (2018). 2017 ACC/AHA/AAPA/ABC/ACPM/AGS/APhA/ASH/ASPC/NMA/PCNA guideline for the prevention, detection, evaluation, and management of high blood pressure in adults: executive summary: a report of the American College of Cardiology/American Heart Association Task Force on clinical practice guidelines. Hypertension.

[26] Sacks FM, Svetkey LP, Vollmer WM (2001). Effects on blood pressure of reduced dietary sodium and the Dietary Approaches to Stop Hypertension (DASH) diet. N Engl J Med.

[27] Levine GN, Bates ER, Bittl JA (2016). 2016 ACC/AHA guideline focused update on duration of dual antiplatelet therapy in patients with coronary artery disease: a report of the American College of Cardiology/American Heart Association Task Force on clinical practice guidelines: an update of the 2011 ACCF/AHA/SCAI guideline for Percutaneous Coronary Intervention, 2011 ACCF/AHA guideline for coronary artery bypass graft surgery, 2012 ACC/AHA/ACP/AATS/PCNA/SCAI/STS guideline for the diagnosis and management of patients with stable ischemic heart disease, 2013 ACCF/AHA guideline for the management of ST-elevation myocardial infarction, 2014 AHA/ACC guideline for the management of patients with non-ST-elevation acute coronary syndromes, and 2014 ACC/AHA guideline on Perioperative Cardiovascular Evaluation and management of patients undergoing noncardiac surgery. Circulation.

[28] Cummings DE, Arterburn DE, Westbrook EO (2016). Gastric bypass surgery vs intensive lifestyle and medical intervention for type 2 diabetes: the CROSSROADS randomised controlled trial. Diabetologia.

[29] Rubino F, Nathan DM, Eckel RH (2016). Metabolic surgery in the treatment algorithm for type 2 diabetes: a joint statement by International Diabetes organizations. Diabetes Care.

[30] Chinnakotla S, Radosevich DM, Dunn TB (2014). Long-term outcomes of total pancreatectomy and islet auto transplantation for hereditary/genetic pancreatitis. J Am Coll Surg.

[31] Pileggi A, Molano RD, Ricordi C, Zahr E, Collins J, Valdes R, Inverardi L (2006). Reversal of diabetes by pancreatic islet transplantation into a subcutaneous, neovascularized device. Transplantation.

[32] Bhatt DL, Kandzari DE, O'Neill WW (2014). A controlled trial of renal denervation for resistant hypertension. N Engl J Med.

[33] Heusser K, Tank J, Engeli S (2010). Carotid baroreceptor stimulation, sympathetic activity, baroreflex function, and blood pressure in hypertensive patients. Hypertension.

[34] Neumann FJ, Sousa-Uva M, Ahlsson A (2019). 2018 ESC/EACTS guidelines on myocardial revascularization. Eur Heart J.

[35] Chambers DC, Cherikh WS, Harhay MO (2019). The International Thoracic Organ Transplant Registry of the International Society for Heart and Lung Transplantation: thirty-sixth adult lung and heart-lung transplantation report-2019; focus theme: donor and recipient size match. J Heart Lung Transplant.

[36] Fourman MM, Saber AA (2012). Robotic bariatric surgery: a systematic review. Surg Obes Relat Dis.

[37] Guber K, Kirtane AJ (2022). Renal sympathetic denervation for hypertension. Kidney Int Rep.

[38] Argenziano M, Katz M, Bonatti J (2006). Results of the prospective multicenter trial of robotically assisted totally endoscopic coronary artery bypass grafting. Ann Thorac Surg.

[39] Currie ME, Romsa J, Fox SA (2012). Long-term angiographic follow-up of robotic-assisted coronary artery revascularization. Ann Thorac Surg.

[40] Keating CL, Dixon JB, Moodie ML, Peeters A, Bulfone L, Maglianno DJ, O'Brien PE (2009). Cost-effectiveness of surgically induced weight loss for the management of type 2 diabetes: modeled lifetime analysis. Diabetes Care.

[41] Fadl Elmula FE, Jin Y, Yang WY (2015). Meta-analysis of randomized controlled trials of renal denervation in treatment-resistant hypertension. Blood Press.

